# Socio‐Economic Status and Non‐Native Species Drive Bird Ecosystem Service Provision in Urban Areas

**DOI:** 10.1111/gcb.70311

**Published:** 2025-07-07

**Authors:** Fabio Marcolin, Stefano Mammola, Riccardo Alba, Pedro Segurado, Luís Reino, Dan Chamberlain

**Affiliations:** ^1^ Forest Research Centre, Associate Laboratory TERRA, School of Agriculture University of Lisbon Lisboa Portugal; ^2^ Department of Life Sciences and System Biology University of Turin Torino Italy; ^3^ CIBIO/InBIO, Centro de Investigação Em Biodiversidade e Recursos Genéticos, School of Agriculture University of Lisbon Lisboa Portugal; ^4^ CIBIO/InBIO, Centro de Investigação Em Biodiversidade e Recursos Genéticos University of Porto Vairão Portugal; ^5^ NBFC, National Biodiversity Future Center Palermo Italy; ^6^ Molecular Ecology Group, Water Research Institute National Research Council Pallanza Italy; ^7^ Finnish Museum of Natural History University of Helsinki Helsinki Finland; ^8^ BIOPOLIS Program in Genomics, Biodiversity and Land Planning CIBIO Vairão Portugal

**Keywords:** alien bird, FUA, functional diversity, Iberian Peninsula, luxury effect, wealth

## Abstract

Areas of higher socio‐economic status within cities often support greater biodiversity than poorer areas, representing a form of environmental injustice. This inequality may result in lower income areas experiencing both lower cultural (e.g., bird aesthetics) and regulating (e.g., pest control) ecosystem service provision. Urban areas are also hotspots for non‐native species, which can alter community functional structure and, consequently, ecosystem service provision. However, the influence of socio‐economic status on services provided by both native and non‐native urban biodiversity remains underexplored. We assessed how functional diversity related to avian cultural and regulating ecosystem services varied along the socio‐economic gradient of functional urban areas (FUAs) in the Iberian Peninsula. Using breeding bird atlases from Spain and Portugal, we characterised bird communities in all FUAs, calculating species richness and functional dispersion based on traits linked to ecosystem services. We used generalised linear mixed models to examine relationships between diversity metrics and median household income. Additionally, we evaluated whether the presence of non‐native species moderated community responses along the gradient. Both cultural and regulating ecosystem services were negatively associated with socio‐economic status, while species richness increased with income. However, invaded communities supported higher species richness and cultural service provision than non‐invaded ones. Our findings reveal a counterintuitive pattern in which ecosystem service provision is higher in lower‐income areas, partly due to non‐native species. These results underscore the need for urban management strategies that simultaneously address socio‐economic and ecological inequalities, while considering the complex roles of non‐native species in shaping urban biodiversity and its benefits.

## Graphical abstract

Birds bring important benefits to people, like pest control and aesthetic enjoyment. We studied birds in cities across Spain and Portugal to see how income levels affect the benefits people get from nature. We found that higher income areas tended to have more bird species but, surprisingly, fewer of these benefits, while places with non‐native birds often had more of them. This shows that both income and the types of birds living in cities can shape how people experience nature in their daily lives.

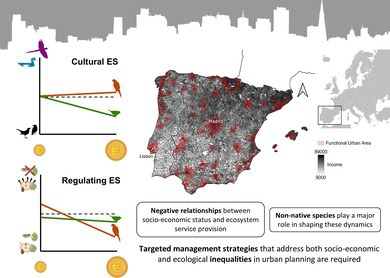

## 1 Introduction

Human pressure through urbanisation has drastically reshaped landscapes across the globe, with 56% of the human population now living within dense urban and peri‐urban areas (United Nations 2018, 2022). It is predicted that by 2050, 68% of the world's population will live in cities (United Nations 2018), posing additional stress on urban ecosystems. Urbanisation has profound effects on the environment (Grimm et al. 2008), and there is a large amount of evidence of negative impacts on biodiversity, typically resulting in a marked decrease in taxonomic diversity with respect to natural or semi‐natural habitats (e.g., Clergeau et al. 2006; Batáry et al. 2018; Mbiba et al. 2021). There are relatively few species able to persist and thrive in urban areas, and these tend to be generalist, widespread species (Evans et al. 2010). Urbanisation thus often leads to biotic homogenisation, whereby communities in urban habitats tend to hold the same species, even across large geographical areas (McKinney 2006; Devictor et al. 2007). Furthermore, cities are hot spots of species introductions (Cardador and Blackburn 2019; Cardador et al. 2022), and often the abundance of non‐native species reaches high levels in urban areas (van Rensburg et al. 2009; Muvengwi et al. 2024).

Although urban ecosystems are highly modified, they can still have biodiversity conservation value (Batáry et al. 2020). Importantly, biodiversity enhances human well‐being in urban areas, with city dwellers typically preferring greener areas with higher species richness that allow for diverse recreational activities and which are positively related to general health and psychological well‐being (Soga et al. 2015; Soga and Gaston 2016; Fischer et al. 2018). However, experiencing urban biodiversity and its associated ecosystem services (e.g., cultural services) is often not equitably shared amongst city inhabitants. This disparity, where higher biodiversity is found in wealthier neighbourhoods, is often referred to as the *luxury effect* (Hope et al. 2003; Leong et al. 2018; Chamberlain et al. 2020) which is recognised as a form of environmental injustice (Chamberlain et al. 2019; Wood et al. 2024). Since Goal 11 of the United Nations sustainable development goals 2030 aims to develop urban planning to reach more inclusive, safer and sustainable cities, and since most studies on the luxury effect have focused on taxonomic diversity (i.e., species richness; Chamberlain et al. 2020), there is a need to understand how socio‐economic context plays a role in shaping biodiversity provision of ecosystem services in urban areas (Reynolds and Howes 2023; Muvengwi et al. 2024). Nonetheless, the definition of urban areas is not universal. As the transition between urban and rural areas is often nuanced (Balta and Atik 2022), there is also a need to understand how biodiversity patterns are shaped along the full urban gradient.

Animals in urban ecosystems provide two main ecosystem services: Cultural and regulating (Millennium Ecosystem Assessment 2005). These services can be indirectly assessed through the functional traits of the species inhabiting urban areas (i.e., urban communities; Behm et al. 2022). For example, functional traits linked to diet or foraging strategy are proxies for regulating ecosystem services such as seed dispersal and pest control (Luck et al. 2012). At the same time, species traits describing bird attractiveness (Echeverri et al. 2020; Santangeli et al. 2023), such as colour diversity or plumage ornaments, can be used as proxy descriptors for cultural services. These include all the non‐material benefits associated with biodiversity such as psychological well‐being deriving from human‐nature connections (Fuller et al. 2007; Dayer et al. 2019), or recreational opportunities through birdwatching (Vallecillo et al. 2019). This approach of using traits as proxy descriptors of ecosystem services has been frequently applied to bird communities for several reasons: (i) birds are present along the whole urban gradient (Callaghan et al. 2019); (ii) detailed traits are available in several published datasets (e.g., Pearman et al. 2014; Tobias et al. 2022; Haukka et al. 2023); and (iii) there is an extensive literature on the links between bird traits and regulating/cultural ecosystem services (e.g., Cameron et al. 2020; Echeverri et al. 2020).

Ecosystem services can be quantified through functional diversity metrics calculated from the species traits of a community (Cadotte et al. 2011; de Bello et al. 2021). For example, different properties of a trait space that aggregate the different traits of a given set of species (e.g., the richness, divergence and regularity components; Mammola et al. 2021) can be used to numerically characterise the amount and distribution of ecosystem services provided by a community. Furthermore, this approach can quantify the impact of different anthropogenic stressors on the trait space itself, and hence assess how ecosystem service provision is affected. For example, non‐native species are well known to preferentially invade disturbed habitats (e.g., urban areas; Cardador and Blackburn 2020), altering the composition of traits of the invaded community and hence the ecosystem functioning and services provided (Ricciardi et al. 2013; Finerty et al. 2016). Recently, Chamberlain et al. (2020) found some support for the luxury effect being more pronounced in non‐native than native species, but they also suggested that the evidence base was not yet sufficient to draw firmer conclusions. Conversely, Villaseñor et al. (2024) found an opposite trend, with non‐native bird species more abundant in lower‐income areas. Nonetheless, there are a few studies considering the influence of socio‐economic context on the ecosystem services provided by urban biodiversity. For urban vegetation, the relationship between regulating ecosystem services and socio‐economic status varies depending on the specific service provided (Aznarez et al. 2023; Muvengwi et al. 2024). For bird communities, the regulating ecosystem services provided in Johannesburg (South Africa) were higher in wealthier neighbourhoods, but cultural ecosystem services were negatively associated with median income (Reynolds and Howes 2023).

We assessed the extent to which socio‐economic status as expressed by median annual household income was associated with species richness and both regulating and cultural ecosystem service provision of urban bird communities across all Functional Urban Areas of the Iberian Peninsula, one of Europe's hotspots for non‐native bird species introductions (Dawson et al. 2017). We hypothesised that species richness and regulating ecosystem services would be greater in higher income areas (i.e., as per the luxury effect), but that the provision of cultural ecosystem services would be lower (as per Reynolds and Howes 2023). Furthermore, we tested whether the presence of non‐native bird species influenced the relationships between socio‐economic status and species richness or ecosystem service provision. In general, we aimed to provide better insights into how socio‐economic factors drive the role of bird communities in ecosystem service provision in urban areas, ideally leading to possible biodiversity management strategies to improve urban equity along with the creation of sustainable urban environments.

## 2 Materials and Methods

### 2.1 Study Area

**FIGURE 1:**
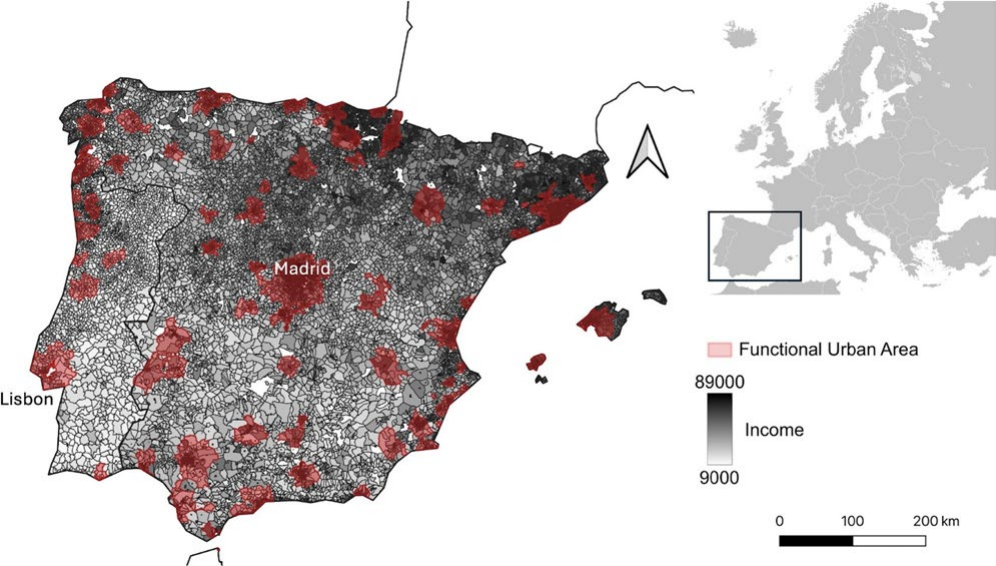
Map of the Iberian Peninsula (Portugal and Spain) and Balearic Islands with Functional Urban Areas (red polygons) and annual median household income per neighbourhood (from light grey to black). Median household income data was not available for white polygons. Cities of Lisbon and Madrid are labelled (capitals of Portugal and Spain respectively). Map lines delineate study areas and do not necessarily depict accepted national boundaries.

We considered all 89 Functional Urban Areas (FUAs, as defined by the European Commission; Dijkstra et al. 2019; Figure 1) of the mainland Iberian Peninsula (including the Balearic Islands but excluding Macaronesia), 76 in Spain and 13 in Portugal. Recently, several international organisations adopted FUAs (Dijkstra et al. 2019; European Union 2021) as their definition for delimiting metropolitan areas. The FUA includes both the city core area and its commuting zone defined by the surrounding areas in which at least 15% of the population commutes to work in the city (Dijkstra et al. 2019; European Union 2021) and thus encompasses a broad range of urban landscapes. Since many lower‐income populations live in commuting zones due to high city housing costs in core FUAs (Blumenberg and Siddiq 2023), the environmental injustice represented by the luxury effect could also apply to these surrounding areas. In Spain, FUAs are distributed along both coastal and interior regions, with significant socio‐economic hubs along the Mediterranean and Atlantic coasts and in Madrid. In contrast, Portugal shows a marked coastal concentration (i.e., Lisbon and Porto). Half of Iberian FUAs have populations between 100,000 and 500,000 inhabitants. Large cities like Madrid, Barcelona, and Lisbon stand out as metropolitan centres, with Madrid being the largest, housing 6 million people.

### 2.2 Bird Data

For Spain, we used data retrieved from the Third Spanish Breeding Bird Atlas (Molina et al. 2022) which recorded occurrences of 450 breeding bird species across ca. five thousand 10 × 10 km UTM grid cells throughout Spain (from 15 April to 15 June in 2014 to 2018). For Portugal, we used data retrieved from the Third Portuguese Breeding Bird Atlas (Equipa Atlas 2022) which recorded occurrences of 227 breeding bird species across almost one thousand 10 × 10 km ETRS cells in continental Portugal (from 30 March to 30 June in 2015 to 2021). For this study, only those UTM/ETRS cells showing a minimum of 50% overlap with Spanish and Portuguese FUAs were included, resulting in a subset of 709 UTM cells in mainland Spain and the Balearic Islands (i.e., Palma de Mallorca), and 111 ETRS cells in continental Portugal. We included occurrences of 276 and 182 species respectively for Spain and Portugal within these FUA grid cells, including only species recorded with at least “possible” breeding status. We later calculated the species richness for each grid cell, treating each one as a community. To assess the effect of rare species, we also explored a more conservative approach, including in the analyses only those species that were observed in at least 1% of atlas grid cells (Virkkala and Lehikoinen 2017), that is, seven occurrences for Spain and one occurrence for Portugal, resulting in 225 and 182 species respectively. Nonetheless, the overall patterns were similar between the two analyses (see Figure S1; Table S1), thus we maintained the first approach in the main results of the paper.

We followed an existing classification (Loiola et al. 2018) to characterise communities in each grid cell in terms of the presence of non‐native species: the community was defined for each grid as *invaded* when at least one non‐native species was present in a community, and defined as *non‐invaded* otherwise. Moreover, to understand the contribution of non‐native species to the functional and phylogenetic space (see section 2.5 below) of an invaded community (Loiola et al. 2018), we examined their native component by excluding non‐native species from the *invaded* communities (*invaded no alien*, where ‘alien’ means non‐native). Furthermore, we followed this approach first including all species (276 and 182 species respectively for Spain and Portugal), and then only those species that were observed in at least 1% of atlas grid cells (225 and 182 species respectively for Spain and Portugal).

### 2.3 Socio‐Economic Status

We used the annual median household income (from now on, median income) at a neighbourhood level as a measure of socio‐economic status (the commonest measure of socio‐economic status in luxury effect studies—Chamberlain et al. 2020). This information was available for the year 2021 in Spain and 2022 in Portugal. We retrieved the data from the respective national statistical institutes: the Instituto Nacional de Estadística (INE) in Spain (Statistic Spain 2024) and the Instituto Nacional de Estatística (INE) in Portugal (Statistic Portugal 2024; Figure 1). To match the bird atlas with the neighbourhood‐level median income, we adjusted the income data to fit the 10 × 10 km grid scale. We did this by calculating a weighted average of the median income, using the area of each neighbourhood polygon that overlapped with the area of each grid cell as the weight. We used this approach to avoid over‐representation of neighbourhoods that overlapped with more than one grid cell (i.e., the contribution of a neighbourhood's income to the overall income of a grid cell was based on its overlapping area with the grid cell). Moreover, this approach allowed for spatial consistency of bird and income data in both countries, enabling a comparison across the study area. Therefore, we did not assign a community to the neighbourhood‐level median income. Instead, we averaged the median income within each predefined grid cell (i.e., 10 × 10 km atlas grid cell), assigning it to the corresponding community occurring within a grid cell. Finally, since the absolute cost of living is lower in Portugal than in Spain (Eurostat 2023), to enable the correct comparison across the study area we standardised the median income separately for each country (i.e., scaled median income). This is necessary in the context of the study, as a given property in Portugal would cost less than exactly the same property in Spain.

### 2.4 Trait Data

We classified the surveyed bird species using a set of 26 functional traits (belonging to nine functional trait groups) retrieved from the literature (Wilman et al. 2014; Storchová and Hořák 2018; Tobias et al. 2022; Santangeli et al. 2023): Five for cultural and 22 for regulating ecosystem services. These traits all have a hypothesised functional link between a given species and the ecosystem service they provide (Swartz et al. 2023), both cultural (e.g., colour diversity as a proxy for aesthetics; Schuetz and Johnston 2019) and regulating (e.g., diet for biological control or seed dispersal; Luck et al. 2012; see Table 1 for details). The traits selected for cultural ecosystem services were: (1) relative tail length (tail length regressed on body mass extracting the residuals; Santangeli et al. 2023); (2) colour diversity; (3) colour elaboration; (4) crest length; and (5) inverse of body mass. For regulating ecosystem services, we selected: (1) degree of social interaction during breeding season (three traits); (2) nesting strategy (five traits); (3) diet during the breeding period (six traits); (4) foraging technique (seven traits); and (5) body mass (Table 1). Apart from tail length, colour diversity, colour elaboration, crest length and body mass (that were continuous variables), the other trait categories were converted to binary variables (i.e., each trait was either present or absent for a given species). As recommended (Palacio et al. 2022), we scaled continuous trait variables to values between 0 and 1, that is, colour diversity, colour elaboration, crest, tail length and body mass (the latter two previously log‐transformed to avoid extreme values; LaBarbera 1989). We subsequently excluded the ‘solitary association during breeding period’ trait variable due to a high correlation with the ‘colonial’ category of the same trait variable (Pearons's *r* = −0.7 and *r* = 0.76 for Spain and Portugal breeding birds respectively, all other Pearson's *r* ≤ ±0.70; Harrison et al. 2018; Dormann et al. 2013; see Tables S2–S5).

**TABLE 1:** Table of bird traits with type (cultural or regulating), detailed description and source, and justification for its inclusion in the analyses.

Trait	Type	Description	Justification
Relative tail length (continuous)	Cultural	Tail length (distance between the tip of longest rectrix and the point at which the two central rectrices protrude) relative to size (body mass) (Tobias et al. 2022)	Ornamental features such as physical structures drive positive visual attraction for birds (Santangeli et al. 2023)
Colour diversity (continuous)	Cultural	Number of cells occupied by colours retrieved from a grid on bird plumage (see Santangeli et al. 2023 for details)	Colour diversity is positively related to bird attractiveness (Schuetz and Johnston 2019)
Colour elaboration (continuous)	Cultural	Average distance between all colours found in a species and the global average colour across all species (see Santangeli et al. 2023 for details)	Colour elaboration, in the form of extreme colours departing from the average, is positively related to bird attractiveness (Schuetz and Johnston 2019)
Crest length (discrete)	Cultural	Four discrete categories: 0 = no crest, 1 = crest follows shape of head, 2 = short crest, and 3 = long crest (see Santangeli et al. 2023 for details)	Ornamental features such as physical structures drive positive visual attraction for birds (Santangeli et al. 2023)
Inverse of body mass (continuous)	Cultural	Based on body mass measured in grams (Tobias et al. 2022). Inverse of body mass was used to avoid correlation with relative tail length and thus retain it in the analyses	Small birds are generally more appreciated and have a strong aesthetic preference by people (Santangeli et al. 2023)
Social degree during breeding season (binomial)	Regulating (grouped)	Three categories with binomial response (0 = no, 1 = yes): solitary, semi‐colonial, colonial (Storchová and Hořák 2018)	Social degree has effects on predation exposure, inter‐specific competition, reproductive outcome and resource use during the breeding period (Swartz et al. 2023).
Nesting strategy (binomial)	Regulating (grouped)	Five categories with binomial response (0 = no, 1 = yes): ground, cavity, open, close to ground, closed‐arborea nest (Storchová and Hořák 2018)	Nesting strategy differs amongst urban habitats based on site availability and affects predation exposure and inter‐specific competition (Swartz et al. 2023).
Diet during breeding period (binomial)	Regulating (grouped)	Six categories with binomial response (0 = no, 1 = yes): granivore, folivore, frugivore, invertebrates, vertebrates, carrion (Storchová and Hořák 2018)	Diet affects competition dynamics within an urban bird assemblage and is related to the habitat (Swartz et al. 2023).
Foraging technique (binomial)	Regulating (grouped)	Eight categories with binomial response (0 = no, 1 = yes): air‐aquatic pursuit, sally, foliage glean, pounce, peck, dig, overturn, probe (Wilman et al. 2014)	Foraging technique gives insights about the ability and plasticity of a given species in resource exploitation in the urban environment (Swartz et al. 2023).
Body mass (continuous)	Regulating	Body mass measured in grams (Tobias et al. 2022)	Body mass is a proxy of ecophysiological conditions, resource exploitation ability, life history and tolerance to disturbance and dispersal ability (Swartz et al. 2023).

*Note:* Categories are not mutually exclusive within a given trait group (i.e., multiple traits can be present). Note that social degree during breeding season, nesting strategy, diet during breeding period and foraging technique categories were grouped to calculate trait dissimilarity amongst species with Gower's distance (Gower 1971).

### 2.5 Estimation of Ecosystem Services Provision

As we were interested in understanding how the presence of non‐native species affected the overall spread and density of traits (i.e., quantifying the ecosystem services provided), we assessed whether *invaded* communities had a higher diversity of traits than *non*‐*invaded* communities (Mammola and Cardoso 2020). Considering that we were working with both phylogenetic data and traits, we measured functional diversity using neighbour‐joining functional trees (Cardoso, Guillerme, et al. 2024). We estimated the divergence component of functional diversity using a measure of functional dispersion of each community (Mammola et al. 2021) and for each ecosystem service provided (i.e., cultural and regulating based on the traits above selected). We calculated functional dispersion with the dispersion function in ‘BAT’ version 2.9.6 (Cardoso, Mammola, et al. 2024), measuring it as the average trait dissimilarity between any two species randomly chosen in a community based on their distances on the functional trees (Cardoso, Guillerme, et al. 2024) representing our species and set of traits. Note that, for this calculation, we excluded communities with less than three species to avoid biased estimations of functional diversity (Mammola et al. 2024), resulting in 709 communities for Spain and 111 for Portugal. Then, we accounted for the phylogenetic signal of each community (Redding et al. 2019), estimating the phylogenetic dispersion of each community's phylogenetic tree through the BAT dispersion function (Cardoso, Guillerme, et al. 2024). We then fitted a linear model to relate functional and phylogenetic dispersion and extracted the model residuals, which represent a phylogenetically corrected measure of functional dispersion for each community (‘observed’ values). Furthermore, to account for the effect of species richness on functional diversity (de Bello et al. 2021; Mammola et al. 2021; Palacio et al. 2022), we generated 999 null distributions randomising the species names in both the functional and phylogenetic trees (each species was assigned a random position in the functional/phylogenetic tree, thereby assuming that a random set of species from the species pool colonised each site at each iteration). For each of the 999 random iterations, we calculated functional and phylogenetic dispersion of communities and used a linear model to obtain the residuals of the fitted relationships between functional and phylogenetic dispersion (‘expected’ values). Subsequently, for each community, we calculated the standard effect size (SES) of the deviation of the phylogenetically corrected functional dispersion (‘observed’ values calculated without randomisations) from the phylogenetically corrected functional dispersion values expected from the random iterations (i.e., the ‘expected’ values generated through null modelling). We calculated SES with the SES function in BAT, with the formula:

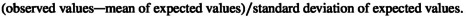




In summary, the final SES values represent the ‘corrected functional dispersion’ (CFD), which accounts for differences in both species' evolutionary history and species richness across communities. These corrected values provide a proxy measure of how communities contribute to ecosystem services: for example, a value of 0 would mean that the ecosystem service provided would be as expected given the species richness of that community; positive and negative values would mean that the ecosystem service provided would be higher or lower than expected given the species richness of that community. We used CFD in all subsequent analyses (see Section S2.5.1 for more details). A visual validation of the procedure is provided in Figures S2–S5.

### 2.6 Statistical Analyses

We assessed whether the socio‐economic status (i.e., luxury effect) affected species richness and the cultural and regulating ecosystem service provision of bird communities in Iberian Peninsula FUAs using mixed effects models (R package nlme version 3.1–163; Pinheiro and Bates 2023). We constructed three models, one for species richness and one each for CFDs based on cultural and regulating ecosystem service provision, specifying an interaction between the scaled median income and community type (*non‐invaded* vs. *invaded*), with an additive effect of country identity (Spain or Portugal) (independent variables). The random component of each model included a random intercept structure represented by the identity of each individual FUA (76 levels in Spain and 13 levels in Portugal; i.e., random = ~1|FUA identity), accounting for the non‐independence of the data as there were multiple grid cells within a given FUA. For species richness, we specified a negative binomial distribution and a log link function (to ensure only positive fitted values). We fitted a negative binomial distribution since the Poisson distribution showed a significant overdispersion (Pearson's Chi‐Squared = 6568.107, *p*‐value < 0.001; check_overdispersion function of the package ‘performance’ version 0.10.9; Lüdecke et al. 2021). For CFD, we specified a Gaussian distribution. Furthermore, to test the species richness and functional dispersion of *invaded* communities accounting only for the native species component (i.e., *invaded no alien*), we tested whether there was a significant difference in species richness and functional dispersion as measured by the CFD values of *invaded* and *invaded no alien* communities for the three models above. This was undertaken using an intercept‐only model, testing whether the difference between the two community types (i.e., CFD of *invaded*–CFD of *invaded no alien*) was significantly different from zero.

We checked model fit with the R package ‘performance’ version 0.10.9 (Lüdecke et al. 2021) by visually inspecting the normality of residuals, heteroskedasticity, and degree of collinearity, finding acceptable model fit in each case. Furthermore, we tested for spatial autocorrelation by using spline correlograms (Bjørnstad and Falck 2001) with 1000 bootstrap resamples (Santana et al. 2017). We inspected the resulting correlogram plots of the full model residuals, and assumed no spatial autocorrelation when 95% confidence intervals included zero (Santana et al. 2017). We found no evidence of any spatial autocorrelation, and thus concluded that our grid cells were spatially independent.

## 3 Results

The number of species found in the FUAs was 282, of which 30 were non‐native (276 and 182 species, of which 27 and 13 were non‐native, for Spain and Portugal respectively). We classified 453 grid cells as *non‐invaded* (437 and 16 for Spain and Portugal respectively) and 367 as *invaded* (272 and 95 for Spain and Portugal respectively). The two countries had 176 species in common, with 109 species unique to Spain and six to Portugal. The species richness per grid cell was (mean ± sd) 66.29 ± 31.14 in Spain and 70.89 ± 19.98 in Portugal. The six most common species shared by both countries, each occurring in at least 84% of the grid cells, were Eurasian blackbird *Turdus merula* , house sparrow *Passer domesticus* , barn swallow *Hirundo rustica* , European serin *Serinus serinus* , common woodpigeon *Columba palumbus* , and European goldfinch *Carduelis carduelis* (for a detailed list of species, see Table S6).

**FIGURE 2:**
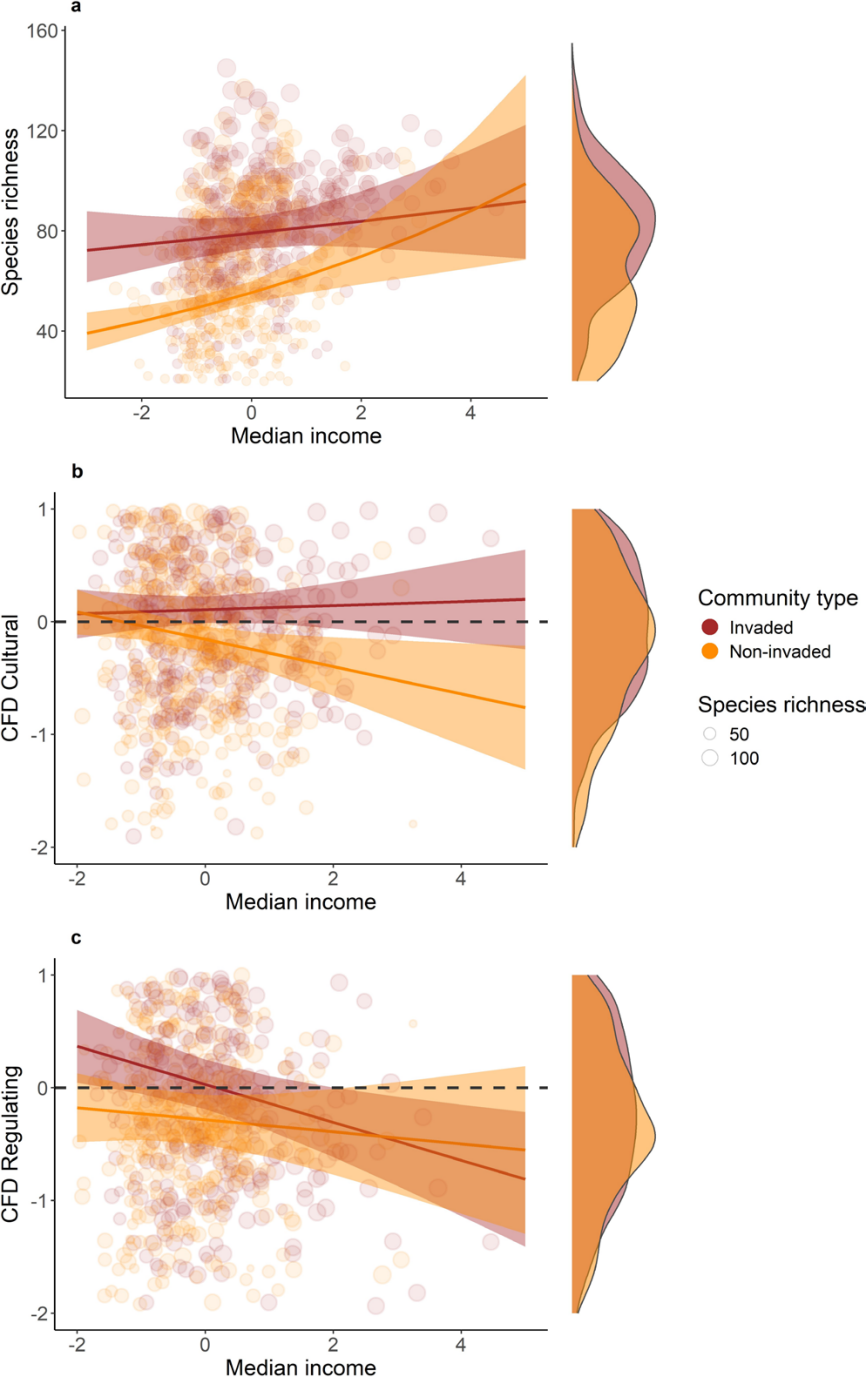
Response of Species richness (a) and CFD [i.e., SES values for corrected functional dispersion, quantification of both Cultural (b) and Regulating (c) ecosystem services provision] to the median income gradient (standardised) in FUAs of Iberian Peninsula. Dot size is proportional to community species richness. The density curves represent the distribution of CFD (both Cultural and Regulating) and species richness distribution. Brown represents the *invaded* communities (i.e., at least one non‐native species was found in the grid cell), and orange represents the *non‐invaded* communities (i.e., no non‐native species were in the grid cell). Underdispersed communities are represented by negative CFD values. Overdispersed communities are represented by positive CFD values.

Species richness increased significantly with increasing scaled median income (χ^2^ = 6.928, *p* < 0.01) and was significantly higher in invaded communities (χ^2^ = 77.831, *p* < 0.001; Table S7; Figure 2a). There was a significant interaction between community type and scaled median income. Species richness increased in both community types, but the increase was relatively slight in *invaded* communities, contrasting with the steep increase in species richness in *non‐invaded* communities along the income gradient. As a result, species richness was higher in *invaded* communities in all but the wealthiest FUAs (χ^2^ = 4.866, *p* = 0.029; Table S7; Figure 2a).

There was a significant difference in CFD for cultural ecosystem services between community types (*F* = 14.210, *p* < 0.001), with higher CFD in *invaded* than in *non‐invaded* communities (i.e., higher cultural ecosystem provision; Table S7; Figure 2b). The two community types differed significantly in their response to the income gradient (interaction *F* = 5.041 *p* = 0.025; Table S7; Figure 2b). In *non‐invaded* communities, CFD significantly decreased with increasing scaled median income (i.e., lower cultural ecosystem provision), but it did not vary significantly in *invaded* communities. When considering only those species that were observed in at least 1% of the atlas grid cells, there was again a significant negative association between CFD and median income in non‐invaded communities, but also a positive significant association between FCD and median income in invaded communities (Table S1; Figure S1). CFD decreased significantly with higher median income in both *non‐invaded* and *invaded* communities for regulating ecosystem services (*F* = 15.014, *p* < 0.001). *Non‐invaded* communities provided generally lower regulating ecosystem services than *invaded* communities (Table S7; Figure 2c).

**FIGURE 3:**
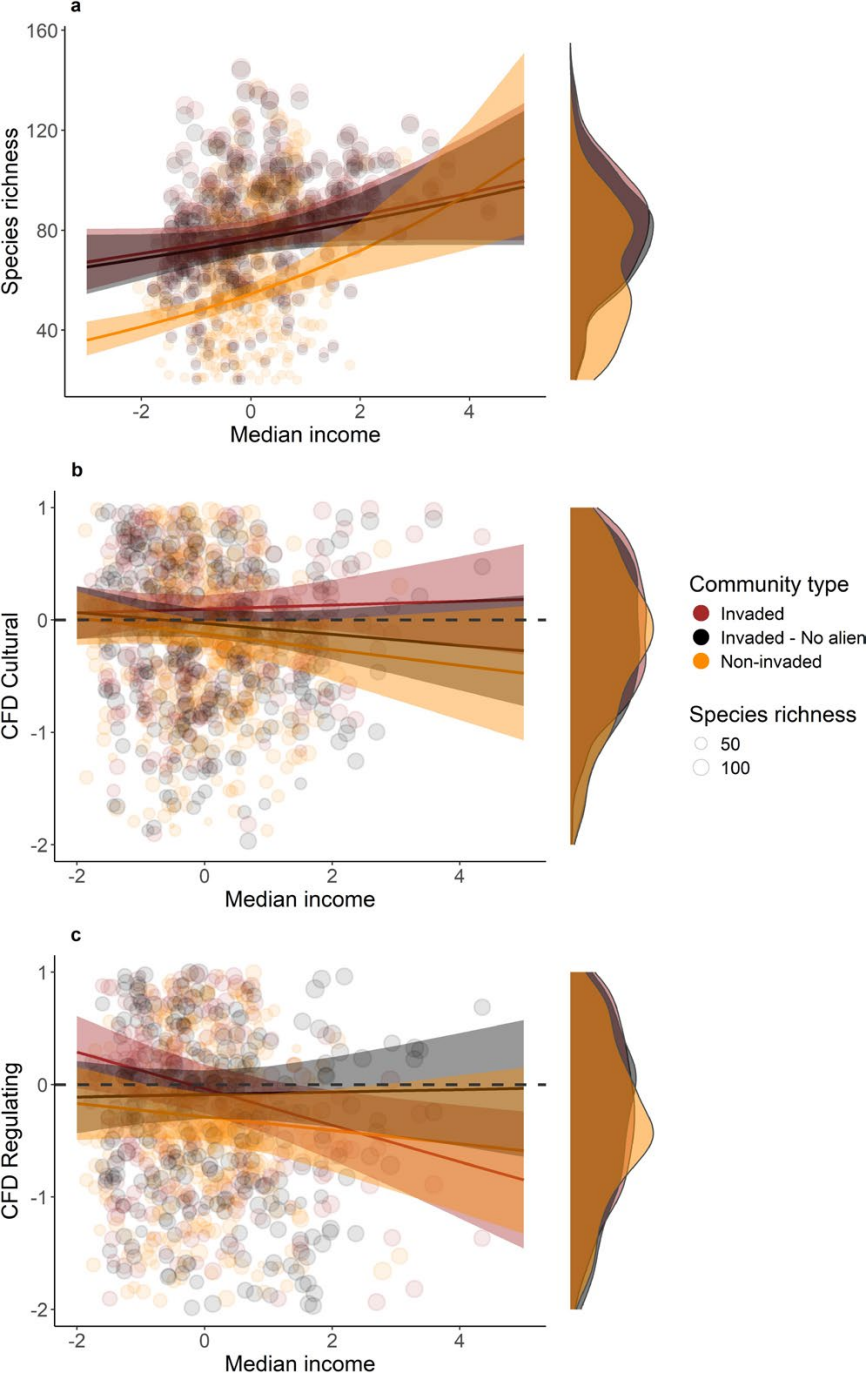
Response of Species richness (a) and CFD [i.e., SES values for corrected functional dispersion, quantification of both Cultural (b) and Regulating (c) ecosystem services provision] to the median income gradient (standardised) in FUAs of Iberian Peninsula. Dot size is proportional to community species richness. The density curves represent the distribution of CFD (both Cultural and Regulating). Brown represents the *invaded* communities (i.e., at least one non‐native species was found in the grid cell), orange represents the *non‐invaded* communities (i.e., no non‐native species were in the grid cell), and black represent the *invaded no alien* communities (i.e., excluding non‐native species from the invaded communities). Underdispersed communities are represented by negative CFD values. Overdispersed communities are represented by positive CFD values.

There was a significant difference between CFD of *invaded* and *invaded no alien* communities for cultural ecosystem services (*F* = 18.389, *p* < 0.001) and for species richness (*χ* ^2^ = 4.300, *p* < 0.001), with a significantly higher CFD and species richness in *invaded* than *invaded no alien* communities (*F* = 4.300, *p* < 0.001; Table S7, Figure 3a,c; Supporting Information S1). There was no significant difference for regulating ecosystem services (*F* = 2.921, *p* = 0.089; Table S7; Figure 3; Supporting Information S1) apart from when we considered only those species that were observed in at least 1% of the atlas grid cells, in which case the *invaded* communities showed higher CFD than *invaded no alien* communities (*F* = 6.298, *p* = 0.013; Table S7; Figure S6c) showing how rare species contribute to the overall regulating ecosystem service provision.

## 4 Discussion

Our study showed that there was a positive association between median income and bird community diversity in urban areas of the Iberian Peninsula, a pattern that has been extensively documented in other regions for a variety of organisms (i.e., luxury effect; Hope et al. 2003; Leong et al. 2018; Chamberlain et al. 2020; Muvengwi et al. 2024). These patterns were evident for both *invaded* and *non‐invaded* bird communities, with non‐native species playing an important role in the bird diversity of *invaded* communities, since *invaded no alien* communities (i.e., *invaded* communities not accounting for the non‐native bird species component) were significantly less diverse. Nonetheless, we did not find any support for the luxury effect being more associated with non‐native species than native species (Chamberlain et al. 2020). Contrary to our expectations, regulating ecosystem services provided by bird communities showed a negative correlation with median income, while the response of cultural ecosystem services was dependent on the bird community type considered, negative association in *non‐invaded* bird communities and a no significant association in *invaded* bird communities. This pattern was more evident when we considered only those species that were observed in at least 1% of the atlas grid cells. Hence, taken together, both analyses suggest opposing responses of the two community types to the median income gradient.

Therefore, the higher cultural ecosystem services of *invaded* bird communities inhabited by non‐native, more colourful, species (e.g., Rose‐ringed parakeet *Psittacula krameri* , Common waxbill *Estrilda astrild* , Mandarin duck *Aix galericulata* ) are seen as more attractive than *non‐invaded* communities. This pattern was also evident when comparing the *invaded* communities with the corresponding *invaded no alien* communities (i.e., *invaded* communities accounting only for native species), with the latter showing significantly lower cultural ecosystem services (i.e., attractiveness). The decrease in cultural ecosystem services with increasing median income in *non‐invaded* communities occurred despite an increase in species richness along the socio‐economic gradient, suggesting that while the number of species increases, they become increasingly aesthetically similar as median income increases.

The decrease in regulating ecosystem services with income in both *invaded* and *non‐invaded* communities could reflect the global pattern of biotic homogenisation in urban areas (McKinney 2006; Devictor et al. 2007). This acts both on functional and phylogenetic diversity, leading to less functionally diverse communities; therefore, decreasing their regulating role on the ecosystem (Morelli et al. 2016). Wealthier neighbourhoods are often characterised by intensively managed green spaces, such as ornamental lawns, and by a higher prevalence of non‐native plants (Lowenstein and Minor 2016). Ornamental lawns with several non‐native plants typically provide limited resources for insects, thereby reducing prey availability for insectivorous bird species (Smith et al. 2015). This would limit the provisioning of regulating ecosystem services (Nyffeler et al. 2018). These results were similar when we considered only those species that were observed in at least 1% of the atlas grid cells, apart from the comparison of corrected functional dispersion of regulating ecosystem services between *invaded* and *non‐invaded* communities, suggesting how rare (native) species play an important role in the regulating ecosystem services provision (Basile 2022; Reynolds and Howes 2023).

There is a bias towards the Global North in luxury effect studies (Chamberlain et al. 2020), and in urban ecology studies in general (Diamant et al. 2025). Conditions in developing countries of the Global South are often very different to those in richer nations in terms of biodiversity, land use, socio‐economic status and income inequality (Reynolds et al. 2021), which may affect responses to socio‐economic gradients. For example, Howes and Reynolds (2021) found no evidence for a luxury effect in terms of bird diversity in Johannesburg, South Africa, in contrast to our results. Furthermore, Reynolds and Howes (2023) found that birds of ‘public interest’ (i.e., a cultural ecosystem service) increased with decreasing socio‐economic status in the same city. The difference between studies likely arises because the aesthetic value (i.e., colourful species; Echeverri et al. 2020; Santangeli et al. 2023) of birds in Southern African urban areas is largely determined by colourful native species (e.g., African Paradise‐flycatcher *Terisphone viridis*, White‐bellied sunbird *Cinnyris talatala* ) that are missing in European urban areas. One key difference between our study and those undertaken in Johannesburg is that we also included the effects of non‐native species in our analyses. In fact, when considering results of cultural ecosystem services in *non‐invaded* bird communities (i.e., communities represented only by native species), our results were similar to those found in Johannesburg (Reynolds and Howes 2023). The wider context (i.e., Global North vs. Global South) is therefore likely important, but the differences between the two studies also highlight the importance of considering species provenance when analysing the luxury effect in terms of ecosystem service provision.

## 5 Conclusions

Our study highlights how socio‐economic status contributes to shaping urban bird community diversity and the ecosystem services it provides in the Iberian Peninsula. We found strong evidence for the luxury effect in these urban areas, with wealthier neighbourhoods supporting higher species richness, both in *non‐invaded* and *invaded* bird communities. However, in terms of cultural ecosystem services, our findings revealed a contrasting relationship with socio‐economic gradients. There was evidence of inequalities between rich and poor areas in ecosystem service provision only for *invaded* communities, thus highlighting the role of non‐native species in mediating the luxury effect and contributing to ecosystem services in wealthier urban areas. Moreover, regulating ecosystem services decreased with increasing median income in both *invaded* and *non‐invaded* communities, likely due to functional homogenisation in wealthier areas.

Urban planning must address the socio‐economic drivers of urban biodiversity and ecosystem service inequality by implementing diverse strategies for managing both native and non‐native bird species (Villaseñor et al. 2024). For example, the creation of green spaces may help to address inequalities in access to taxonomic diversity in economically disadvantaged areas. Furthermore, management strategies, such as the promotion of native vegetation and the enhancement of structural complexity can have multiple benefits, such as maintaining functional diversity and encouraging species that are more sensitive to impacts of urbanisation (Mbiba et al. 2021; Rogers et al. 2021; Muvengwi et al. 2022). Our results add a new dimension to the luxury effect research base by showing that assessing ecosystem services and including non‐native species in analyses of biodiversity‐socio‐economic gradients can provide a contrasting picture to the typical positive association between taxonomic diversity and wealth status. Importantly, our results show that taxonomic biodiversity and ecosystem service provision can have opposing responses to the socio‐economic gradient. Strategies to enhance taxonomic diversity in poorer areas therefore also need to maintain the higher regulating and cultural ecosystem services already present in those areas. Furthermore, our results suggest that non‐native bird species contribute to ecosystem services independently of socio‐economic status in urban areas. Thus, despite their potential damaging effects on native biodiversity (Pyšek et al. 2020), non‐native species do appear to have benefits for people in the highly modified urban landscape.

Addressing the environmental injustice represented by the luxury effect represents a significant challenge if we are to account for ecosystem service provision and non‐native species as well as taxonomic diversity. We call for similar studies to address different aspects of biodiversity simultaneously in order to determine if our findings from the Iberian Peninsula are applicable more broadly. Any strategies that are developed to confront the above issues need to be assessed by including the costs and benefits for the human population. For example, the creation of green spaces in poorer urban areas may result in green gentrification whereby the environmental improvement results in increased property prices, ultimately causing the displacement of part of the population with little impact on the luxury effect (Anguelovski et al. 2022). Balancing these ecological and social considerations is essential for fostering sustainable and inclusive urban environments, highlighting the need to view urban areas as valuable spaces for biodiversity conservation, complementing pristine and protected areas (Batáry et al. 2020), but also as vital elements for the quality of life of the people who live there.

## Author Contributions

**Fabio Marcolin:** conceptualization, data curation, formal analysis, investigation, methodology, visualization, writing – original draft, writing – review and editing. **Stefano Mammola:** formal analysis, methodology, writing – original draft, writing – review and editing. **Riccardo Alba:** visualization, writing – original draft, writing – review and editing. **Pedro Segurado:** writing – original draft, writing – review and editing. **Luís Reino:** writing – original draft, writing – review and editing. **Dan Chamberlain:** conceptualization, formal analysis, investigation, methodology, visualization, writing – original draft, writing – review and editing.

## Conflicts of Interest

The authors declare no conflicts of interest.

## Supporting information


**Figure S1.** Response of species richness (a) and CFD [i.e., SES values for corrected functional dispersion, quantification of both cultural (b) and regulating (c) ecosystem services provision] to the median income gradient (standardised) in FUAs of Iberian Peninsula, including in the analyses only those species that were observed in at least 1% of atlas grid cells (Virkkala and Lehikoinen 2017) for example, seven occurrences for Spain and one occurrence for Portugal. Dot size is proportional to community species richness. The density curves represent the distribution of CFD (both cultural and regulating). Brown represents the *invaded* communities (i.e., at least one non‐native species was found in the grid cell), and orange represents the *non‐invaded* communities (i.e., no non‐native species were in the grid cell). Underdispersed communities are represented by negative CFD values. Overdispersed communities are represented by positive CFD values.
**Table S1**. LMM outputs for effects on CFD (SES values for corrected functional dispersion) excluding the species that occurred in less than 1% of the grid cells (i.e., at least seven occurrences for breeding bird species in Spain). (a) Effects of median income (scaled), community type (non‐invaded, invaded), country identity (Portugal, Spain), and two‐way interactions between median income and community type; (b) the intercept‐only model on the corrected functional dispersion difference between *Invaded* and *Invaded no alien* bird communities.
**Figure S2**. Plots reporting the correlation between the species richness and the corrected functional dispersion for cultural ecosystem services in Iberian Peninsula (both Portugal and Spain) accounting for all the breeding bird species (*r* = 0.240; *p* < 0.001). *r* is the Pearson’s regression value; *p* represents the *p*‐value of the regression.
**Figure S3**. Plots reporting the correlation between the species richness and the corrected functional dispersion for regulating ecosystem services in Iberian Peninsula (both Portugal and Spain) accounting for all the breeding bird species (*r* = 0.011; *p* < 0.001). *r* is the Pearson’s regression value; *p* represents the *p*‐value of the regression.
**Figure S4**. Plots reporting the correlation between the phylogenetic diversity and the observed residuals of functional dispersion (i.e., residuals from a linear regression between the functional and phylogenetic diversity) for cultural ecosystem services in Iberian Peninsula (both Portugal and Spain) accounting for all the breeding bird species (*r* = 0.000; *p* = 1). *r* is the Pearson’s regression value; p represents the *p*‐value of the regression
**Figure S5**. Plots reporting the correlation between the phylogenetic diversity and the observed residuals of functional dispersion (i.e., residuals from a linear regression between the functional and phylogenetic diversity) for regulating ecosystem services in Iberian Peninsula (both Portugal and Spain) accounting for all the breeding bird species (*r* = 0.000; *p* = 1). *r* is the Pearson’s regression value; *p* represents the *p*‐value of the regression.
**Figure S6**. Response of species richness (a) and CFD [i.e., SES values for corrected functional dispersion, quantification of both cultural (b) and regulating (c) ecosystem services provision] to the median income gradient (standardised) in FUAs of Iberian Peninsula, including in the analyses only those species that were observed in at least 1% of atlas grid cells (Virkkala and Lehikoinen 2017) that is, seven occurrences for Spain and one occurrence for Portugal. Dot size is proportional to community species richness. The density curves represent the distribution of CFD (both Cultural and Regulating). Brown represents the *invaded* communities (i.e., at least one non‐native species was found in the grid cell), orange represents the *non‐invaded* communities (i.e., no non‐native species were in the grid cell), and black represent the *invaded no alien* communities (i.e., excluding non‐native species from the invaded communities). Underdispersed communities are represented by negative CFD values. Overdispersed communities are represented by positive CFD values.
**Table S7**. LMM outputs for effects on CFD (SES values for corrected functional dispersion) including all the breeding bird species (i.e., main results from the manuscript). (a) Effects of median income (scaled), community type (non‐invaded, invaded), country identity (Portugal, Spain), and two‐way interactions between median income and community type; (b) the intercept‐only model on the corrected functional dispersion difference between *Invaded*—*Invaded no alien* bird communities.

**Data S1.** Tables S2–S6.

## Data Availability

The data and R scripts that support the findings of this study are openly available in Dryad Digital Repository at https://doi.org/10.5061/dryad.vx0k6dk3z. Raw species occurrence data from national breeding bird atlases are available upon request from SEO/BirdLife at https://atlasaves.seo.org/ (Spain) and Sociedade Portuguesa para o Estudo das Aves (SPEA) at https://www.listavermelhadasaves.pt/atlas/ (Portugal). Median household income data were obtained from Instituto Nacional de Estadística (INE) at https://www.ine.es (Spain) and Instituto Nacional de Estatística (INE) at https://www.ine.pt (Portugal).
