# Supplementary material for: Socio‐Economic Status and Non‐Native Species Drive Bird Ecosystem Service Provision in Urban Areas
Source: Glob Chang Biol. 2025 Jul 7;31(7):e70311. doi: 10.1111/gcb.70311 (PMC12230893; doi:10.1111/gcb.70311)
Supplement: Supplementary file 1 — Figure S1. Response of species richness (a) and CFD [i.e., SES values for corrected functional dispersion, quantification of both cultural (b) and regulating (c) ecosystem services provision] to the median income gradient (standardised) in FUAs of Iberian Peninsula, including in the analyses only those species that were observed in at least 1% of atlas grid cells (Virkkala and Lehikoinen 2017) for example, seven occurrences for Spain and one occurrence for Portugal. Dot size is proportional to community species richness. The density curves represent the distribution of CFD (both cultural and regulating). Brown represents the invaded communities (i.e., at least one non‐native species was found in the grid cell), and orange represents the non‐invaded communities (i.e., no non‐native species were in the grid cell). Underdispersed communities are represented by negative CFD values. Overdispersed communities are represented by positive CFD values. Table S1. LMM outputs for effects on CFD (SES values for corrected functional dispersion) excluding the species that occurred in less than 1% of the grid cells (i.e., at least seven occurrences for breeding bird species in Spain). (a) Effects of median income (scaled), community type (non‐invaded, invaded), country identity (Portugal, Spain), and two‐way interactions between median income and community type; (b) the intercept‐only model on the corrected functional dispersion difference between Invaded and Invaded no alien bird communities. Figure S2. Plots reporting the correlation between the species richness and the corrected functional dispersion for cultural ecosystem services in Iberian Peninsula (both Portugal and Spain) accounting for all the breeding bird species (r = 0.240; p < 0.001). r is the Pearson’s regression value; p represents the p‐value of the regression. Figure S3. Plots reporting the correlation between the species richness and the corrected functional dispersion for regulating ecosystem services in Iberian [file GCB-31-e70311-s002.docx]

**Supporting Information for**

**Socio-economic status and non-native species drive bird ecosystem service provision in urban areas**

**Fig. S1** Response of Species richness (a) and CFD [i.e. SES values for corrected functional dispersion, quantification of both Cultural (b) and Regulating (c) ecosystem services provision] to the median income gradient (standardised) in FUAs of Iberian Peninsula, including in the analyses only those species that were observed in at least one percent of atlas grid cells (Virkkala & Lehikoinen, 2017) i.e. seven occurrences for Spain and one occurrence for Portugal. Dot size is proportional to community species richness. The density curves represent the distribution of CFD (both Cultural and Regulating). Brown represents the *invaded* communities (i.e. at least one non-native species was found in the grid cell), and orange represents the *non-invaded* communities (i.e. no non-native species were in the grid cell). Underdispersed communities are represented by negative CFD values. Overdispersed communities are represented by positive CFD values.
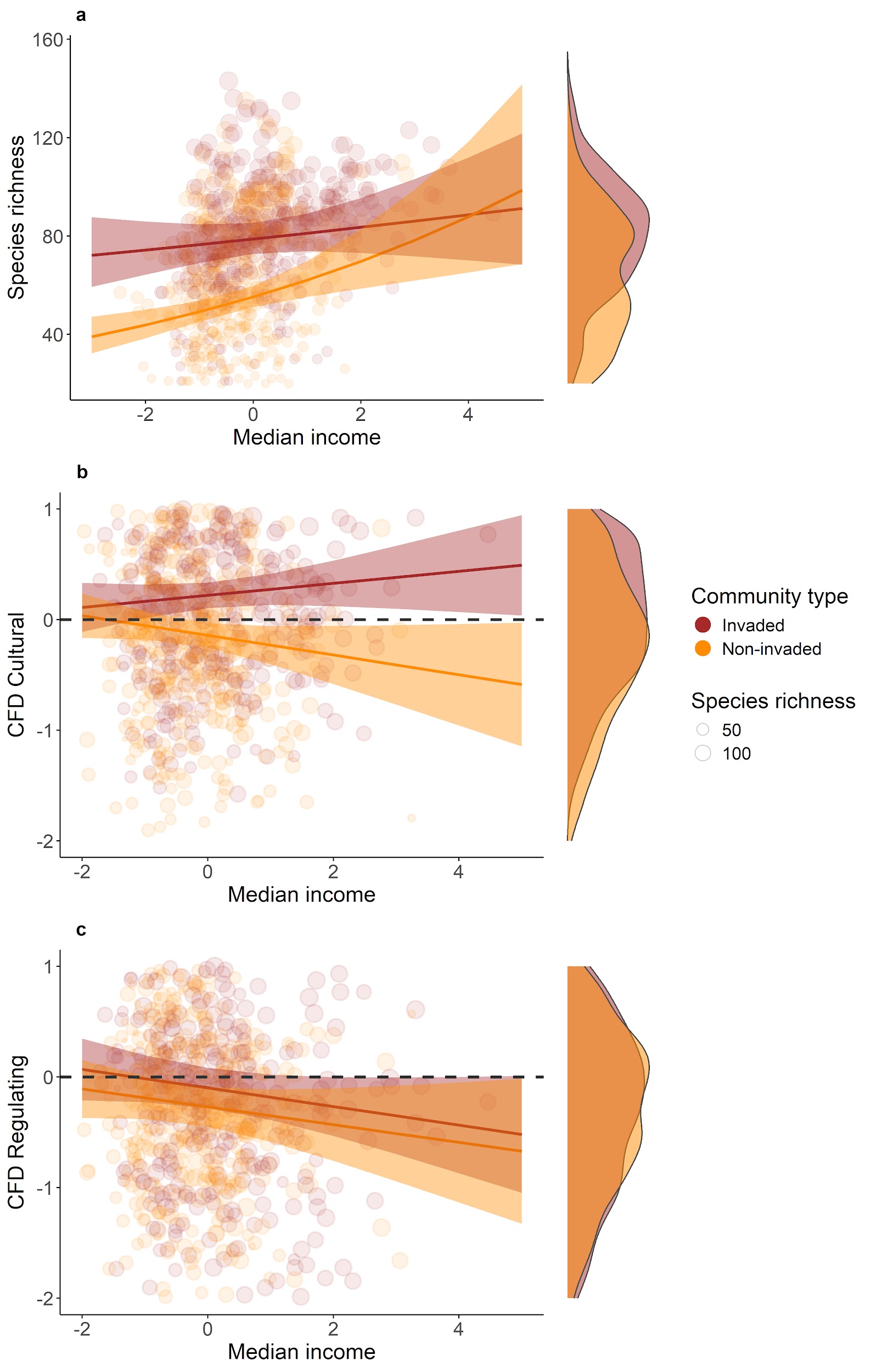


**Table S1** LMM outputs for effects on CFD (SES values for corrected functional dispersion) excluding the species that occurred in less than one percent of the grid cells (i.e. at least 7 occurrences for breeding bird species in Spain). (a) Effects of median income (scaled), community type (non-invaded, invaded), country identity (Portugal, Spain), and two-way interactions between median income and community type; (b) the intercept only model on the corrected functional dispersion difference between *Invaded* - *Invaded no alien* bird communities.


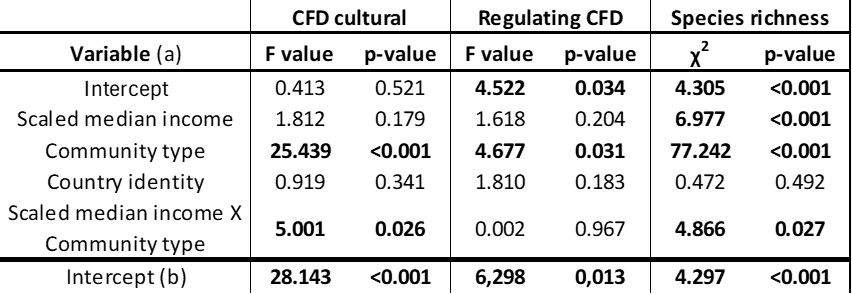


Variables, F values and relative p values are reported for the overall effects. Significant results are reported in bold.

**2.5.1. Estimation of ecosystem services provision – detailed procedure**

There are several methods for mathematically representing the trait space of communities, such as ordinations, functional trees and multidimensional spaces (Mammola et al., 2021). Here, we used a functional tree (Petchey & Gaston, 2002), as this method provides the most direct comparison with taxonomic and phylogenetic diversity measures (Mammola et al., 2021; Cardoso et al., 2024a; see below). We built the functional tree representing our species and set of traits through the tree.build function from the R package ‘BAT’ version 2.9.6 (Cardoso et al., 2015; Cardoso et al., 2024b), choosing the neighbour-joining algorithm (Saitou & Nei, 1987; Cardoso et al., 2024a). Since our trait matrix included both continuous and binary traits, we calculated trait dissimilarity among species with Gower’s distance (Gower, 1971), using the gawdis function from the ‘gawdis’ R package version 0.1.5 (de Bello et al., 2021). This approach allows weights to be assigned automatically to multiple traits that reflect similar functions, thereby avoiding a disproportionate influence of certain traits on the estimation of Gower’s distance. Therefore, we grouped each variable that reflected the same trait information (e.g., the six variables of ‘diet’ were treated as part of the same trait), giving an optimised weight to the five trait groups (de Bello et al., 2021). We kept traits reflecting cultural ecosystem services separated since each trait represented a distinct characteristic of a species (specific grouping of traits is given in Table 1 of the main manuscript). As we were interested in understanding how the presence of non-native species affected the overall spread and density of traits (i.e. quantifying the ecosystem services provided), we assessed whether invaded communities had a higher diversity of traits than non-invaded communities (Mammola & Cardoso, 2020). Thus, we estimated the divergence component of functional diversity using a measure of functional dispersion of each community (Mammola et al., 2021) and for each ecosystem service provided (i.e. cultural and regulating based on the traits above selected). We calculated functional dispersion with the dispersion function in BAT, measuring it as the average dissimilarity between any two species randomly chosen in a community based on their distances on the functional tree (Webb et al., 2002; Cardoso et al., 2024a). Note that, for this calculation, we excluded communities with a species richness < 3 to avoid biassed estimations of functional diversity (Mammola et al., 2024), resulting in 709 communities for Spain and 111 for Portugal.

Since life-history traits (e.g. body mass) are generally strongly phylogenetic conserved, we accounted for the phylogenetic signal of each community (Redding et al., 2019). We downloaded 1000 phylogenetic trees for our set of species based on the Hackett backbone (Hackett et al. 2008) from BirdTree (Jetz et al. 2012). We then selected a median phylogenetic tree, namely the tree with the smallest Kendall–Colijn distance from the full set of 1000 trees (Jombart et al., 2017), using the R package treespace (function medTree with default settings; Jombart et al., 2017). We estimated the phylogenetic dispersion of each community’s phylogenetic tree through the BAT dispersion function (Cardoso et al., 2024b). We then fit a linear model to relate functional and phylogenetic dispersion and extracted the model residuals, which represent a phylogenetically corrected measure of functional dispersion for each community. In this context, positive residuals indicate communities where functional diversity exceeds expectations based on the phylogenetic signal, suggesting a greater diversity of functional traits than predicted by the phylogenetic relationships among species in the community. Conversely, negative residuals indicate communities with lower-than-expected functional diversity, where functional traits are more constrained by phylogenetic relationships. It is important to note that a phylogenetic regression, such as a phylogenetic generalized least squares model, could not be applied in our study because our analyses were conducted at the community level rather than at the species level.

Functional diversity metrics are also often strongly correlated with the species richness of the studied communities (de Bello et al., 2021; Mammola et al., 2021; Palacio et al., 2022). To account for the effect of species richness on functional diversity, we generated 999 null distributions randomising the species names in both the functional and phylogenetic trees (each species was assigned a random position in the functional/phylogenetic tree, thereby assuming that a random set of species from the species pool colonised each site at each iteration). For each of the 999 random iterations, we calculated functional and phylogenetic dispersion of communities and used a linear model to obtain the residuals of the fitted relationships between functional and phylogenetic dispersion (“expected” values). Subsequently, for each community, we calculated the standard effect size (SES) of the deviation of the phylogenetically corrected functional dispersion (“observed values” calculated without randomisations) from the phylogenetically corrected functional dispersion values expected from the random iterations (i.e. the “expected values” generated through null modelling). We calculated SES with the ses function in BAT, with the formula:

(observed values – mean of expected values) / standard deviation of expected values

Positive SES values reflect an overdispersed community (namely, one that shows higher cultural/regulating ecosystem service provision than expected given the number of species within the community) and negative SES values reflect an underdispersed community (i.e., lower cultural/regulating ecosystem service provision than expected given the number of species within the community). In summary, the final SES values represent the 'corrected functional dispersion' (CFD), which accounts for differences in both species' evolutionary history and species richness across communities. This corrected values provides proxy measure of how communities contribute to ecosystem services (i.e. a value of 0 would mean that the ecosystem service provided would be as expected given the species richness of that community). We used CFD in all subsequent analyses. A visual validation of the procedure is shown in Supporting Information Figures S2-S5.

**2.5.2. Contextualising the Corrected Functional Diversity (CFD)**

Phylogenetic and functional diversity are widely employed as complementary measures to understand community assembly and ecosystem functioning (Saavedra et al. 2014; Whitfeld et al. 2014; Matuoka et al. 2020). Despite strong phylogenetic signal is often observed in ecological traits, suggesting that closely related species tend to exhibit similar functional characteristics (de Bello et al., 2017; Cadotte et al., 2019), this pattern shows incongruencies (Cadotte et al., 2019). Functional and phylogenetic dispersion patterns frequently diverge (Cadotte et al., 2019), and several studies show that measured traits only partially account for the phylogenetic structure observed in communities (Li et al., 2017). In our study, we sought the least complicated method to explicitly account for phylogenetic conservatism in functional diversity (therefore at community level). While methods such as HMSC can jointly estimate phylogenetic signals across traits and communities (Tikhonov et al., 2019), their implementation is not congruent with the estimation of functional diversity indices. Other studies assess phylogenetic signal on individual PCoA trait axes (using Blomberg’s K; Mazel et al., 2019) or on singular traits (Li et al., 2017), which provides descriptive insights into trait conservatism, but does not correct functional diversity metrics accordingly. In contrast, our proposed metric, Corrected Functional Dispersion (CFD), uses a regression-based approach to isolate the residual variation in functional dispersion not explained by phylogenetic dispersion across communities. This residual, corrected for species richness via a null model approach (de Bello et al., 2021; Palacio et al., 2022), provides a community-level measure of trait dispersion that is independent of both shared evolutionary history and richness-driven artefacts (see Fig. S2-S5). By focusing on these residuals, CFD captures cases of functional overdispersion (i.e. communities provide a greater breadth of ecological functions than expected from their phylogeny), and underdispersion (i.e. trait diversity is constrained by evolutionatìry history). This approach allows us to test hypotheses about community assembly more directly, especially in cases where phylogeny and trait data are only partially aligned (de Bello et al., 2017; Li et al., 2017). Moreover, it offers a more reliable alternative to the "phylogenetic gambit" (i.e. the assumption that phylogenetic diversity can reliably serve as a surrogate for functional diversity; Mazel et al., 2018).

We provide the R script to show how community-level functional and phylogenetic dispersions are computed, how regression residuals are extracted, and how final SES values (CFD) are derived using the BAT R package (Supporting Information 3).


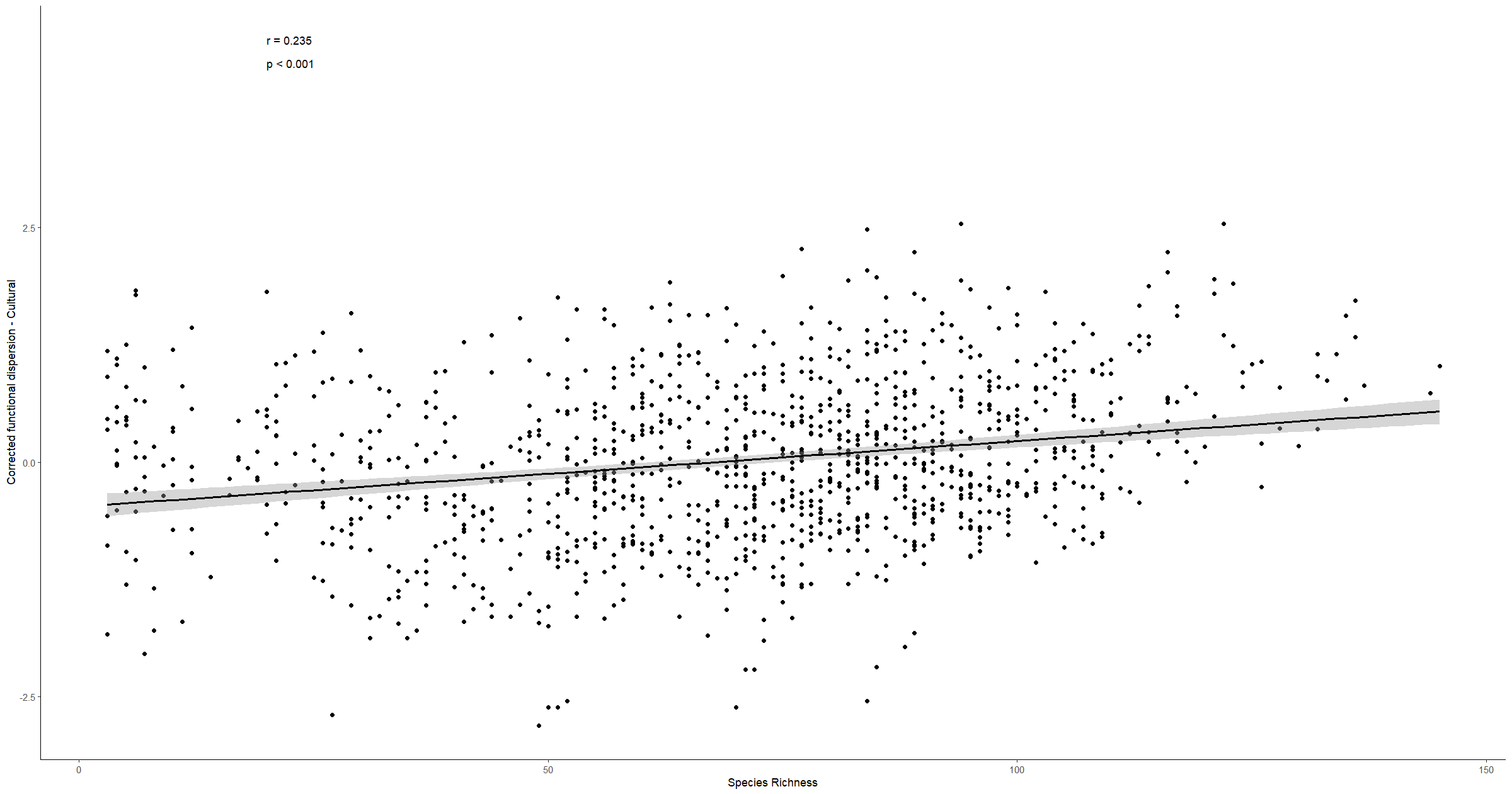


**Fig. S2** Plots reporting the correlation between the Species Richness and the corrected functional dispersion for cultural ecosystem services in Iberian Peninsula (both Portugal and Spain) accounting for all the breeding bird species (r = 0.240; p < 0.001). r is the Pearson’s regression value; p represents the p-value of the regression.

**Fig. S3** Plots reporting the correlation between the Species Richness and the corrected functional dispersion for regulating ecosystem services in Iberian Peninsula (both Portugal and Spain) accounting for all the breeding bird species (r = 0.011; p < 0.001). r is the Pearson’s regression value; p represents the p-value of the regression.
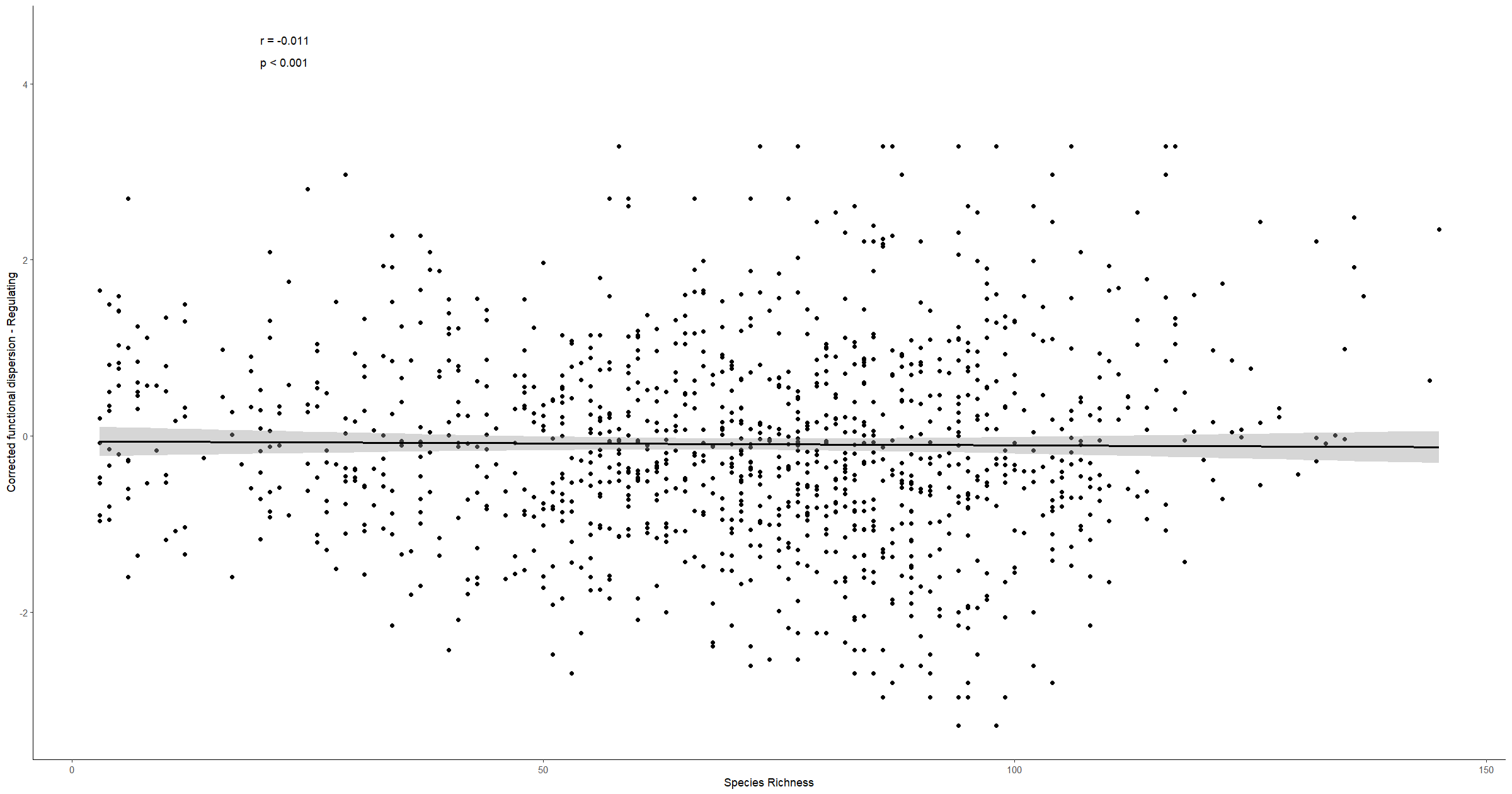


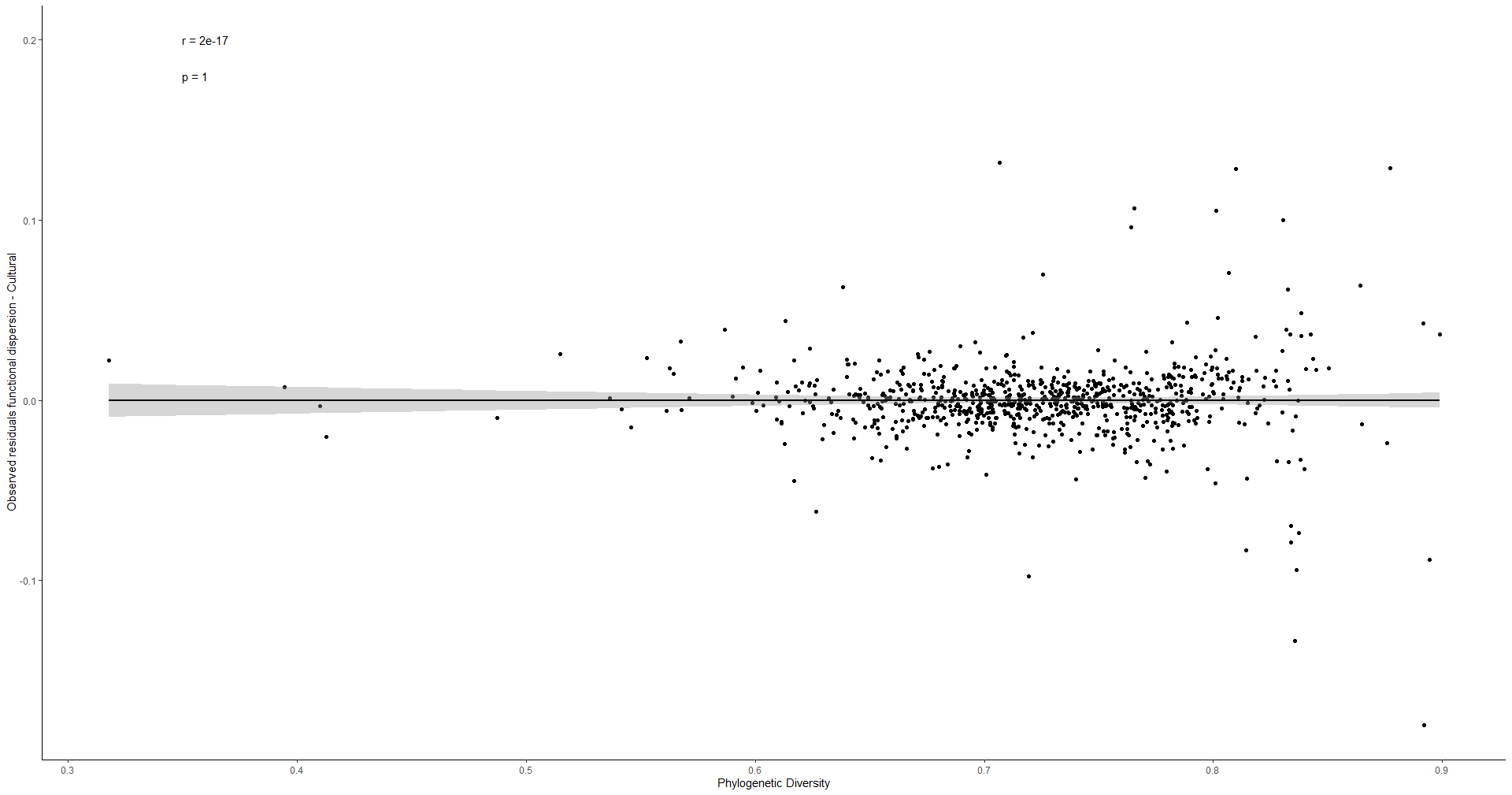


**Fig. S4** Plots reporting the correlation between the phylogenetic diversity and the observed residuals of functional dispersion (i.e. residuals from a linear regression between the functional and phylogenetic diversity) for cultural ecosystem services in Iberian Peninsula (both Portugal and Spain) accounting for all the breeding bird species (r = 0.000; p = 1). r is the Pearson’s regression value; p represents the p-value of the regression

**Fig. S5** Plots reporting the correlation between the phylogenetic diversity and the observed residuals of functional dispersion (i.e. residuals from a linear regression between the functional and phylogenetic diversity) for regulating ecosystem services in Iberian Peninsula (both Portugal and Spain) accounting for all the breeding bird species (r = 0.000; p = 1). r is the Pearson’s regression value; p represents the p-value of the regression.
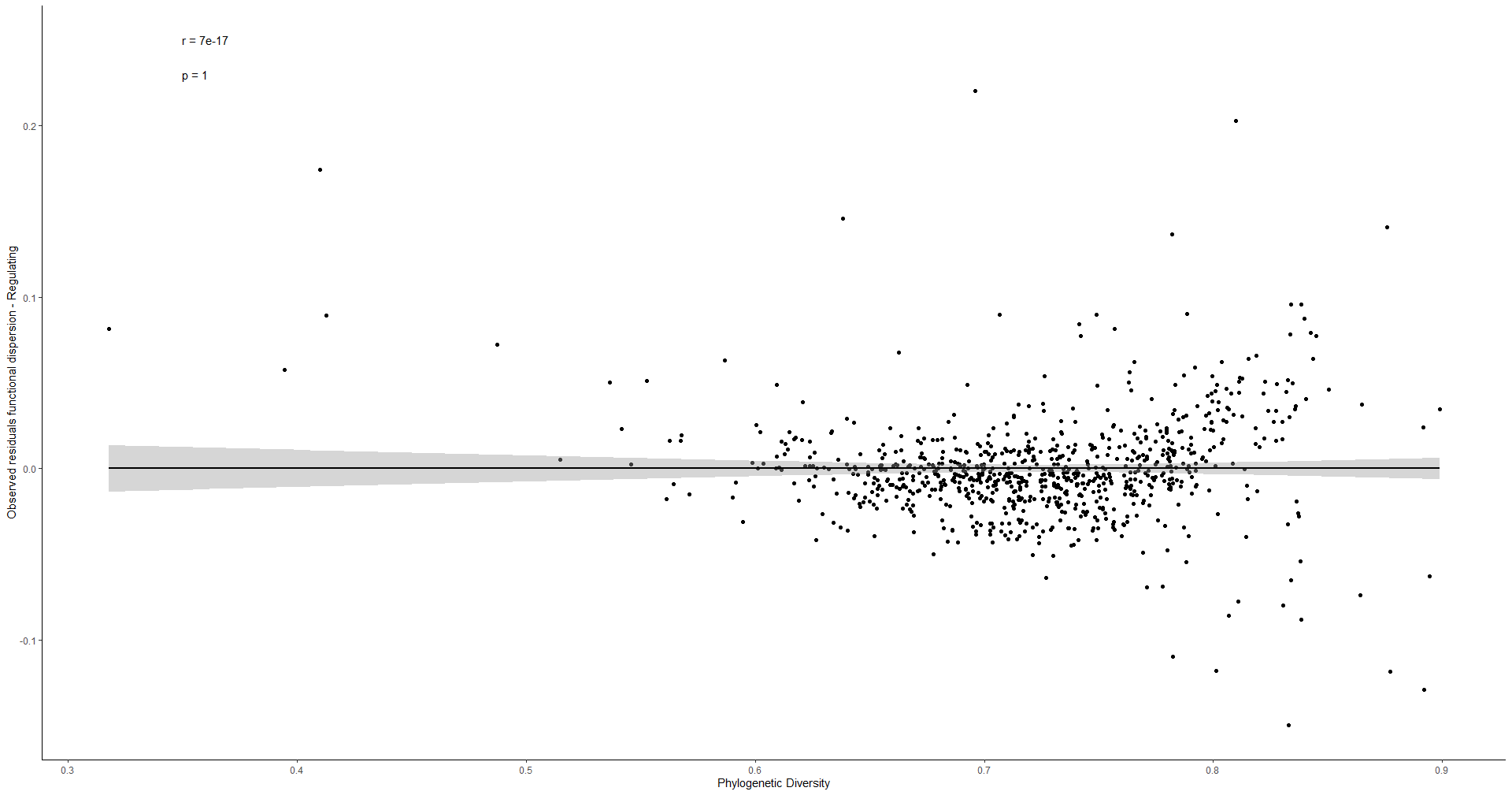


**Fig. S6** Response of Species richness (a) and CFD [i.e. SES values for corrected functional dispersion, quantification of both Cultural (b) and Regulating (c) ecosystem services provision] to the median income gradient (standardised) in FUAs of Iberian Peninsula, including in the analyses only those species that were observed in at least one percent of atlas grid cells (Virkkala & Lehikoinen, 2017) i.e. seven occurrences for Spain and one occurrence for Portugal. Dot size is proportional to community species richness. The density curves represent the distribution of CFD (both Cultural and Regulating). Brown represents the *invaded* communities (i.e. at least one non-native species was found in the grid cell), orange represents the *non-invaded* communities (i.e. no non-native species were in the grid cell), and black represent the *invaded no alien* communities (i.e. excluding non-native species from the invaded communities). Underdispersed communities are represented by negative CFD values. Overdispersed communities are represented by positive CFD values.
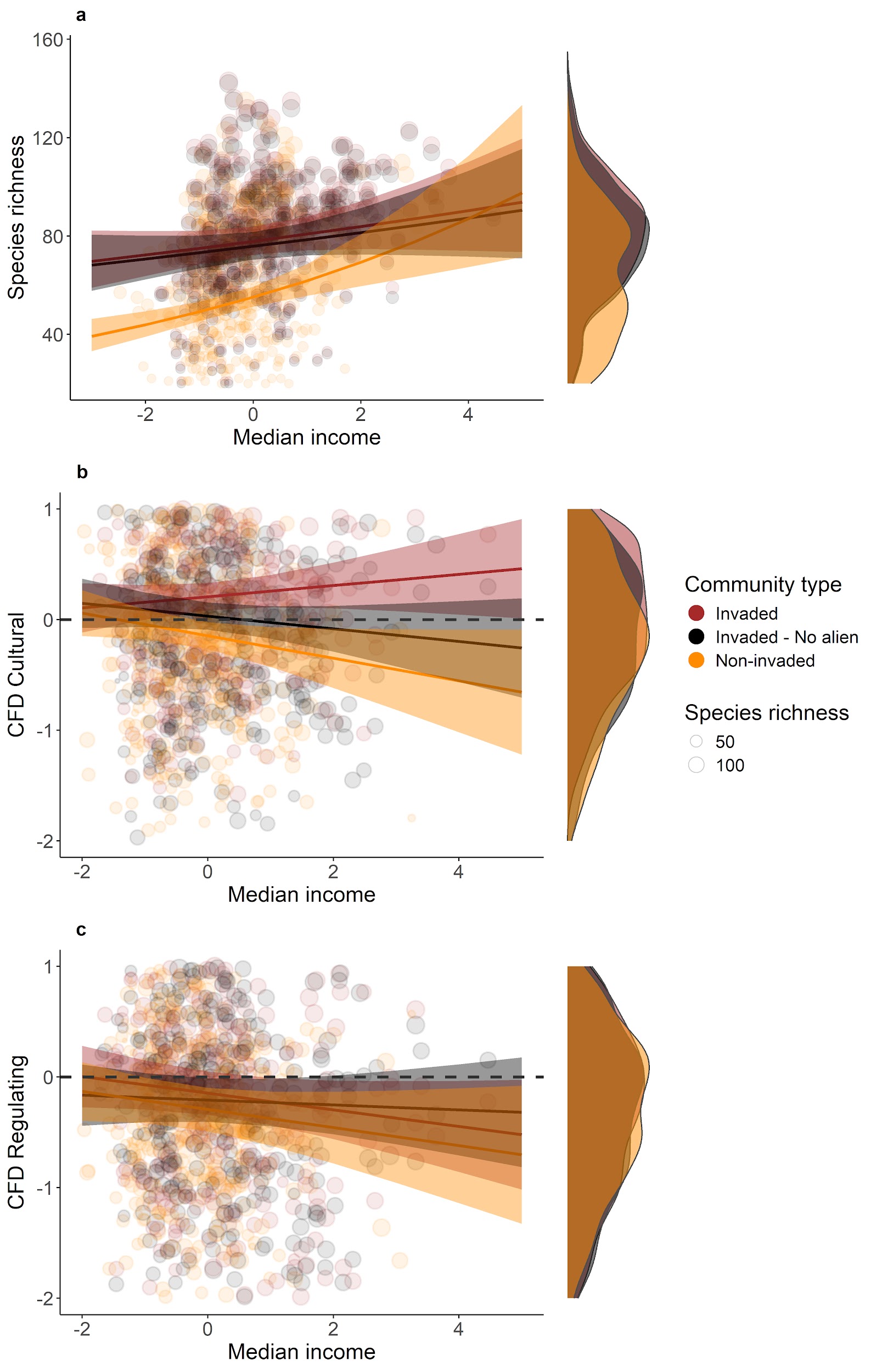


**Table S7** LMM outputs for effects on CFD (SES values for corrected functional dispersion) including all the breeding bird species (i.e. main results from the manuscript). (a) Effects of median income (scaled), community type (non-invaded, invaded), country id entity (Portugal, Spain), and two-way interactions between median income and community type; (b) the intercept only model on the corrected functional dispersion difference between *Invaded* - *Invaded no alien* bird communities.


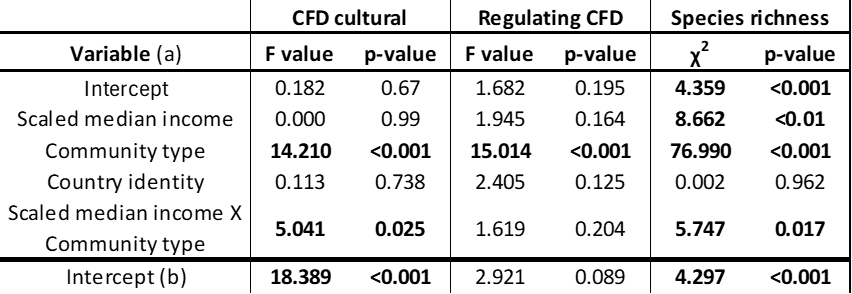


Variables, F values and relative p values are reported for the overall effects. Significant results are reported in bold.

**References**

Cadotte, M. W., Carboni, M., Si, X., & Tatsumi, S. (2019). Do traits and phylogeny support congruent community diversity patterns and assembly inferences?. Journal of Ecology, 107(5), 2065-2077.

Cardoso, P., Guillerme, T., Mammola, S., Matthews, T. J., Rigal, F., Graco‐Roza, C., ... & Carlos Carvalho, J. (2024a). Calculating functional diversity metrics using neighbor‐joining trees. Ecography, e07156.

Cardoso, P., Mammola, S., Rigal, F. Carvalho, J.C. (2024b). BAT: Biodiversity assessment tools. R package, https:// CRAN.R- project.org/ package= BAT (https://doi.org/

Cardoso, P., Rigal, F., Carvalho, J.C. (2015). BAT–Biodiversity Assessment Tools, an R package for the measurement and estimation of alpha and beta taxon, phylogenetic and functional diversity. Methods in Ecology and Evolution, 6(2), 232-236. (https://doi.org/10.1111/2041-210X.12310)

de Bello, F., Šmilauer, P., Diniz‐Filho, J. A. F., Carmona, C. P., Lososová, Z., Herben, T., & Götzenberger, L. (2017). Decoupling phylogenetic and functional diversity to reveal hidden signals in community assembly. Methods in Ecology and Evolution, 8(10), 1200-1211.

de Bello, F., Carmona, C.P., Dias, A.T., Götzenberger, L., Moretti, M., Berg, M. P. (2021). Handbook of trait-based ecology: from theory to R tools. Cambridge University Press.

Gower, J.C. (1971). A general coefficient of similarity and some of its properties. Biometrics, 857-871.

Hackett, S. J., Kimball, R. T., Reddy, S., Bowie, R. C., Braun, E. L., Braun, M. J., ... & Yuri, T. (2008). A phylogenomic study of birds reveals their evolutionary history. science, 320(5884), 1763-1768.

Jetz, W., Thomas, G. H., Joy, J. B., Hartmann, K., & Mooers, A. O. (2012). The global diversity of birds in space and time. Nature, 491(7424), 444-448.

Jombart, T., Kendall, M., Almagro‐Garcia, J., & Colijn, C. (2017). treespace: Statistical exploration of landscapes of phylogenetic trees. Molecular ecology resources, 17(6), 1385-1392.

Li, D., Ives, A. R., & Waller, D. M. (2017). Can functional traits account for phylogenetic signal in community composition?. New Phytologist, 214(2), 607-618.

Mammola, S., & Cardoso, P. (2020). Functional diversity metrics using kernel density n‐dimensional hypervolumes. Methods in Ecology and Evolution, 11(8), 986-995.

Mammola, S., Carmona, C. P., Guillerme, T., & Cardoso, P. (2021). Concepts and applications in functional diversity. Functional Ecology, 35(9), 1869-1885.

Mammola, S., Graco‐Roza, C., Ballarin, F., Hesselberg, T., Isaia, M., Lunghi, E., Mouron, S., Pavlek, M., Tolve, M., Cardoso, P. (2024). Functional convergence underground? The scale‐dependency of community assembly processes in European cave spiders. Global ecology and biogeography, 33(6), e13840. https://doi.org/10.1111/geb.13840

Matuoka, M. A., Benchimol, M., de Almeida-Rocha, J. M., & Morante-Filho, J. C. (2020). Effects of anthropogenic disturbances on bird functional diversity: A global meta-analysis. Ecological Indicators, 116, 106471.

Mazel, F., Pennell, M. W., Cadotte, M. W., Diaz, S., Dalla Riva, G. V., Grenyer, R., ... & Pearse, W. D. (2018). Prioritizing phylogenetic diversity captures functional diversity unreliably. Nature communications, 9(1), 2888.

Palacio, F. X., Callaghan, C. T., Cardoso, P., Hudgins, E. J., Jarzyna, M. A., Ottaviani, G., ... & Mammola, S. (2022). A protocol for reproducible functional diversity analyses. Ecography, 2022(11), e06287.

Petchey, O. L., & Gaston, K. J. (2002). Extinction and the loss of functional diversity. Proceedings of the Royal Society of London. Series B: Biological Sciences, 269(1501), 1721-1727.

Redding, D. W., Pigot, A. L., Dyer, E. E., Şekercioğlu, Ç. H., Kark, S., & Blackburn, T. M. (2019). Location-level processes drive the establishment of alien bird populations worldwide. Nature, 571(7763), 103-106.

Saavedra, F., Hensen, I., Beck, S. G., Böhning-Gaese, K., Lippok, D., Töpfer, T., & Schleuning, M. (2014). Functional importance of avian seed dispersers changes in response to human-induced forest edges in tropical seed-dispersal networks. Oecologia, 176, 837-848.

Saitou, N., & Nei, M. (1987). The neighbor-joining method: a new method for reconstructing phylogenetic trees. Molecular biology and evolution, 4(4), 406-425.

Webb, C. O., Ackerly, D. D., McPeek, M. A., & Donoghue, M. J. (2002). Phylogenies and community ecology. Annual review of ecology and systematics, 33(1), 475-505.

Whitfeld, T. J., Lodge, A. G., Roth, A. M., & Reich, P. B. (2014). Community phylogenetic diversity and abiotic site characteristics influence abundance of the invasive plant Rhamnus cathartica L. Journal of Plant Ecology, 7(2), 202-209.
